# Overexpression of PRDM16 improves muscle function after rotator cuff tears

**DOI:** 10.1016/j.jse.2024.05.037

**Published:** 2024-07-19

**Authors:** He Zhang, Aboubacar Wague, Agustin Diaz, Mengyao Liu, Luke Sang, Alex Youn, Sankalp Sharma, Nesa Milan, Hubert Kim, Brian Feeley, Xuhui Liu

**Affiliations:** a Department of Physical Education, Central South University, Changsha, Hunan, China; b Department of Veterans Affairs, San Francisco Veterans Affairs Medical Center, San Francisco, CA, USA; c Department of Orthopedic Surgery, University of California, San Francisco, San Francisco, CA, USA

**Keywords:** Rotator cuff tears, beige fat, fatty infiltration, fibrosis, fibro-adipogenic progenitor, muscle atrophy

## Abstract

**Background:**

Muscle atrophy, fibrosis, and fatty infiltration are commonly seen in rotator cuff tears (RCTs), which are critical factors that directly determine the clinical outcomes for patients with this injury. Therefore, improving muscle quality after RCT is crucial in improving the clinical outcome of tendon repair. In recent years, it has been discovered that adults have functional beige/brown adipose tissue (BAT) that can secrete batokines to promote muscle growth. PRDM16, a PR-domain-containing protein, was discovered with the ability to determine the brown fat cell fate and stimulate its development. Thus, the goal of this study was to discover the role of PRDM16 in improving muscle function after massive tendon tears using a transgenic mouse model with an elevated level of PRDM16 expression.

**Methods:**

Transgenic aP2-driven PRDM16-overexpressing mice and C57BL/6J mice underwent unilateral supraspinatus (SS) tendon transection and suprascapular nerve transection (TTDN) as described previously (n = 8 in each group). DigiGait was performed to evaluate forelimb function at 6 weeks post the TTDN injury. Bilateral SS muscles, interscapular brown fat, epididymal white fat, and inguinal beige fat were harvested for analysis. The expression of PRDM16 in adipose tissue was detected by Western blot. Masson Trichrome staining was conducted to evaluate the muscle fibrosis, and Oil Red O staining was used to determine the fat infiltration. Muscle fiber type was determined by major histocompatibility complex (MHC) expression via immunostaining. All data were presented in the form of mean ± standard deviation. *t* test and 2-way analysis of variance was performed to determine a statistically significant difference between groups. Significance was considered when *P* < .05.

**Results:**

Western blot data showed an increased expression of PRDM16 protein in both white and brown fat in PRDM16-overexpressing mice compared with wild-type (WT) mice. Even though PRDM16 overexpression had no effect on increasing muscle weight, it significantly improved the forelimbs function with longer brake, stance, and stride time and larger stride length and paw area in mice after RCT. Additionally, PRDM16-overexpressing mice showed no difference in the amount of fibrosis when compared to WT mice; however, they had a significantly reduced area of fatty infiltration. These mice also exhibited abundant MHC-IIx fiber percentage in the supraspinatus muscle after TTDN.

**Conclusion:**

Overexpression of PRDM16 significantly improved muscle function and reduced fatty infiltration after rotator cuff tears. Promoting BAT activity is beneficial in improving rotator cuff muscle quality and shoulder function after RCT.

**Level of evidence:**

Basic Science Study; Histology/Gene Expression; In-Vivo Animal Model

Rotator cuff tears are among the most common upper extremity injuries affecting 20%−40% of the general population.^13,35^ With our aging population, the prevalence of this condition is expected to increase. Although it is ideal to repair immediately after injury, many patients are asymptomatic for 1–3 years before symptoms emerge.^19,25,46^ This leads to the development and accumulation of secondary muscle degeneration including muscle atrophy, fibrosis, and fatty infiltration, which are critical components in determining clinical outcomes of tendon repair. Failed repair leads to a heavy societal burden, a high cost of disease, and a significantly diminished quality of life.^8,47^ In light of the stagnating improvement in clinical outcomes, it is important to identify new ways to recover muscle quality after rotator cuff tears, thus improving clinical outcomes.^24^

In recent years, studies have uncovered a resident stem cell population known as fibroadipogenic progenitors (FAPs) that serve as important regulators in the promotion of muscle growth and regeneration as well as the development of intramuscular fibrosis and fatty infiltration.^14,15,37,45^ In order to promote healthy muscle regeneration, FAPs go through a complex pathway and differentiate into beige/brown adipose tissue (BAT). Besides the thermogenic function of BAT, it also serves an important role in promoting muscle growth through the secretion of batokines.^20,38,39^ This improves proliferation of skeletal muscle stem cells and their differentiation into myoblast and myotubes. Further understanding of the mechanisms that regulate the pathway of BAT differentiation opens a large door in regenerative medicine.

PRDM16, a PR-domain-containing protein, drives the molecular phenotype of brown fat cells, and knockdown of this gene ablates the genetic program of brown fat.^11,17,31^ Interestingly, FAPs contain receptors for PRDM16, which when bound, causes differentiation into BAT-FAPs.^49^ Receptors for this protein are also located in intramuscular white adipose tissue and leads to the conversion of white fat into brown fat.^9^ This could potentially improve outcomes in rotator cuff tear patients, as fatty infiltration and muscle quality are significant predictors for successful clinical outcomes.^50^

In this study, we hypothesized that overexpression of PRDM16 would lead to improved muscle function after rotator cuff injury as measured by gait analysis. Secondarily, we aimed to see if PRDM16 overexpression improves muscle quality through decreased intramuscular fibrosis and fatty infiltration.

## Methods

### Mice and rotator cuff injury

PRDM16-overexpressing mice were kindly provided by the Kajimura Lab (University of California, San Francisco, CA, USA). For transgenic mice, the complete PRDM16 cDNA was cloned 3′ to the 5.4-kb aP2 promoter/enhancer and the human growth hormone polyadenylation site was inserted 3′ to the cDNA.^33^ C57BL/6J mice wild type (WT) mice were purchased from the Jackson Laboratories. All of the mice used for the experiments were 4–6-month-old males (n = 8 in each group). The mice were maintained on a standard chow diet with 12-hour light and dark cycles. All mice underwent unilateral right supraspinatus tendon transection and suprascapular nerve transection (TTDN), as described in Liu et al.^22^ The contralateral side was used as control. Overexpression was confirmed via Western blot (n = 4) and analyzed via ImageJ.

### Western blot

Proteins from epididymal white adipose tissue (white fat), epididymal beige adipose tissue (beige fat), and interscapular brown adipose tissue (brown fat) from PRDM16-overexpressing and wildtype (WT) mice were extracted using T-PER tissue protein extraction reagent (cat. no. 78510; Thermo Scientific, Waltham, MA, USA) with protease inhibitor cocktail tablets (cat. no. 04693116001; Roche, Indianapolis, IN, USA) and phosphatase inhibitor (cat. no. A32957; Thermo Scientific). Protein concentration were measures using Pierce BCA protein assay kit (cat. no. 23225; Thermo Scientific). The samples were then heated up with loading buffer at 98°C for 5 minutes, separated on 4%−12% NuPAGE Bis-Tris gels (Thermo Scientific), and then transferred onto polyvinylidene fluoride membranes (no. 88518; Thermo Scientific). Membranes were blocked in Tris-buffered saline with 0.1% Tween-20 (TBS-T buffer) containing 5% skim milk at room temperature for 30 minutes, followed by incubation with rabbit polyclonal anti-PRDM16 primary antibody (cat. no. 720206; Thermo Scientific) at 4°C overnight. Membranes were washed with TBS-T buffer 3 times (5 minutes each time) and incubated with antirabbit secondary antibodies for 1 hour at room temperature. Immunoreactive bands were visualized using the LI-COR Odyssey Platform. Glyceraldehyde 3-phosphate dehydrogenase was used as the loading control. Quantification was conducted using ImageJ. The differences were displayed with the relative value, which was normalized to the sham control.

### Gait analysis

Gait data were collected using the DigiGait Imaging System developed by Mouse Specifics Inc. that allows for quantitative measures of motion kinematics, motor function, and coordination. Evaluations were conducted preoperatively and 6 weeks post rotator cuff injury. All mice walked at 10 cm/s for 10 seconds on the DigiGait system. Stride length, stance width, peak paw area, stride frequency, and stance time are the parameters to assess forelimb function. Stride length was defined as the distance covered between the stance and swing phases. Stance width was defined as the distance between the center of each forelimb paw. Peak paw area was defined as area of paw at full stance. These factors allowed us to assess shoulder function as described in previous studies.^10^

### Muscle harvesting and histology

At 6 weeks, mice were killed and the bilateral supraspinatus muscles, interscapular brown fat, epididymal white fat, and inguinal beige fat were harvested. Following wet weight measurement, muscle samples were mounted with 5% tragacanth gum and flash-frozen in liquid nitrogen–cooled isopentane and sectioned (10-μm) with a cryostat. Sections at muscle belly for each sample were stained with Masson Trichrome kit (American MasterTech, Lodi, CA, USA) and Oil Red O kit (Sigma Aldrich Inc., St. Louis, MO, USA) according to the manufacturer’s instruction to assess the fibrosis (fibrotic area / total muscle area) and fatty infiltration (at area / total muscle area) in supraspinatus muscles. Quantification was conducted using ImageJ using cross-sectional area macros. The slides were reviewed by 2 blinded reviewers. At least 5 randomly chosen locations were counted for each slide.

For muscle fiber type analysis, slides were fixed in 4% paraformaldehyde, washed with 0.1% phosphate-buffered saline–Triton X-100 (Sigma Aldrich Inc., St. Louis, MO, USA), and blocked in 2% bovine serum albumin for 1 hour. Samples were then incubated with major histocompatibility complex I (MHC-I) (DSHB BA-D5 1:600 bovine anti-mouse), MHC-IIa (DSHB SC-71 1:600 bovine anti-mouse), MHC-IIb (DSHB BF-F3 1:100 bovine anti-mouse), and MHC-IIx (DSHB 6H1 1:50 rabbit anti-mouse) at 4°C overnight.^12^ Slides were then rinsed and incubated with secondary antibodies for goat anti-mouse IgG2b Alexa Fluor 488 (cat. no. 115-547-187,1:200; Jackson ImmunoResearch Laboratories Inc., West Grove, PA, USA), goat ant-mouse IgG1 DyLight 405 (cat. no. 115-477-185,1:200; Jackson ImmunoResearch Laboratories Inc.), goat anti-mouse IgM Alexa Fluor 594 (cat. no. 115-585-020,1:200; Jackson ImmunoResearch Laboratories Inc.) at 1 hour at room temperature. Quantification was conducted using ImageJ.

### Statistical analysis

Student *t* test and 2-way analysis of variance (ANOVA) were used to analyze differences between groups with regard to gait parameters, wet muscle weight, fibrosis, fatty infiltration, and muscle fiber type, where appropriate. Significance was defined as *P* < .05.

## Results

### PRDM16 expression predominately in white fat and brown fat

**Figure 1:**
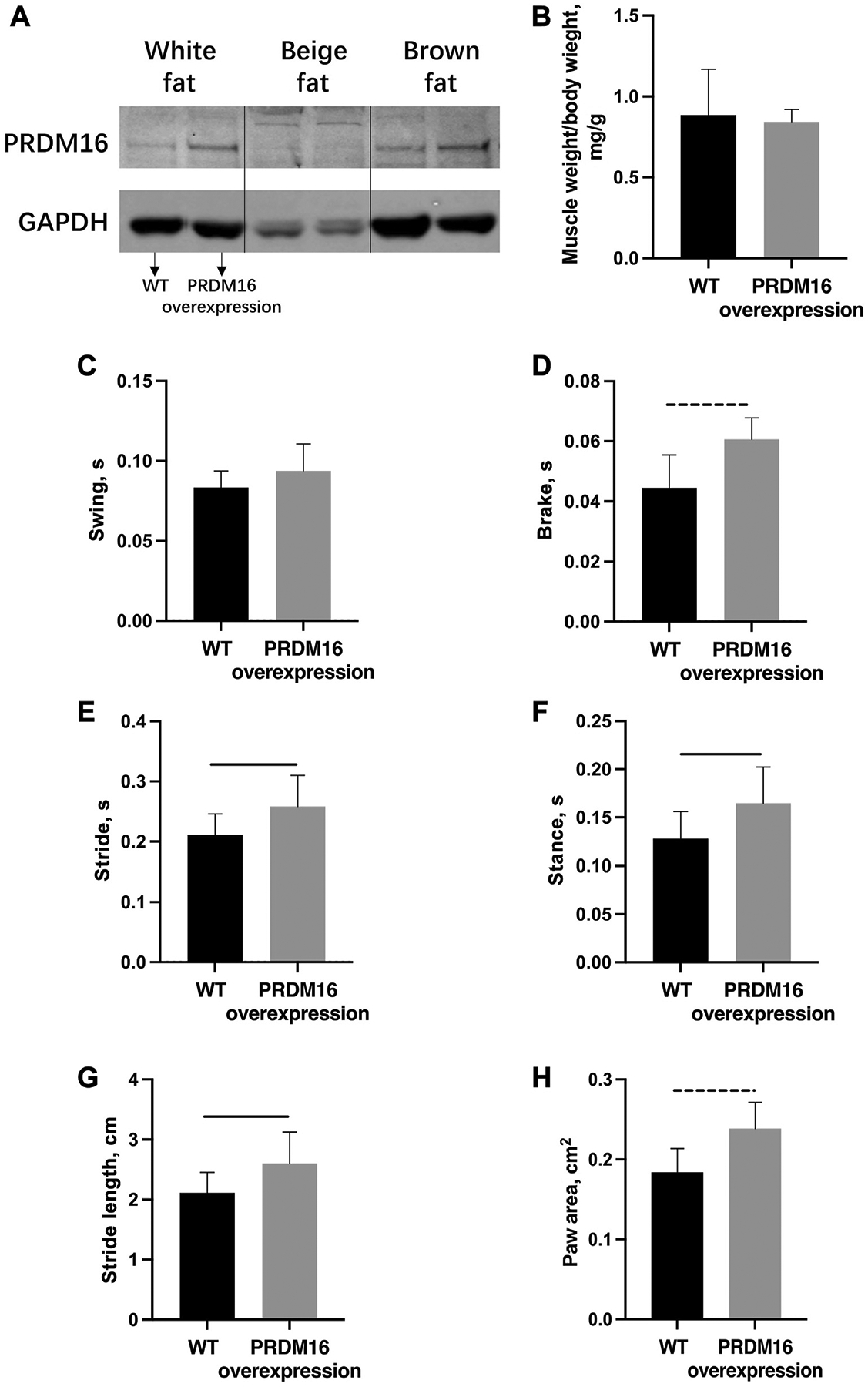
(**A**) Western blot analysis of PRDM16 in adipose tissue showed increased PRDM16 protein expression in both white and brown fat in PRDM16-overexpressing mice compared with WT mice. (**B**) There was no significant difference of relative supraspinatus muscle weight (normalized to body weight) in PRDM16-overexpressing and WT mice at 6 weeks after TTDN. (**C-H**) PRDM16-overexpressing mice have significantly improved upper limb function seen through multiple parameters in DigiGait, such as stride length and time, compared with WT mice at 6 weeks after TTDN. *Solid lines* indicate *P* < .05 and *dashed lines* indicate *P* < .01. *WT*, wild type; *TTDN*, right supraspinatus tendon transection and suprascapular nerve transection.

Western blot data showed increased expression of PRDM16 protein in both white (2.549 ± 0.677–fold, *P* = .020) and brown fat (1.963 ± 0.609–fold, *P* = .009) in PRDM16-overexpressing mice when compared to WT mice (Fig. 1, A). Wet weights of supraspinatus muscle when normalized to body weight exhibited no difference between PRDM16-overexpressing mice and WT mice (0.843 ± 0.078 mg/g vs. 0.865 ± 0.315 mg/g, *P* = .854; Fig. 1, B).

### Overexpression of PRDM16 improves shoulder function after rotator cuff injury

Mice with overexpression of the *PRDM16* gene exhibited improved shoulder function based on gait analysis. At 6 weeks post TTDN injury, PRDM16-overexpressing mice demonstrated significantly longer brake (0.061 ± 0.007 seconds vs. 0.045 ± 0.012 seconds, *P* = .008), stride (0.259 ± 0.052 seconds vs. 0.206 ± 0.032 seconds, *P* = .031), and stance times (0.164 ± 0.038 seconds vs. 0.124 ± 0.026 seconds, *P* = .026) than WT mice (Fig. 1, C). Furthermore, PRDM16-overexpressing mice showed significantly longer stride length (2.60 ± 0.526 cm vs. 2.05 ± 0.312 cm, *P* = .027) and significantly larger paw area (0.239 ± 0.033 cm^2^ vs. 0.184 ± 0.031 cm^2^, *P* =.004) than WT mice (Fig. 1, C). There was no significant change in swing time (0.094 ± 0.017 seconds vs. 0.083 ± 0.011 seconds, *P* = .135) between the PRDM16-overexpressing and WT groups (Fig. 1, C).

### PRDM16 overexpression does not impact the amount of muscle fibrosis

**Figure 2:**
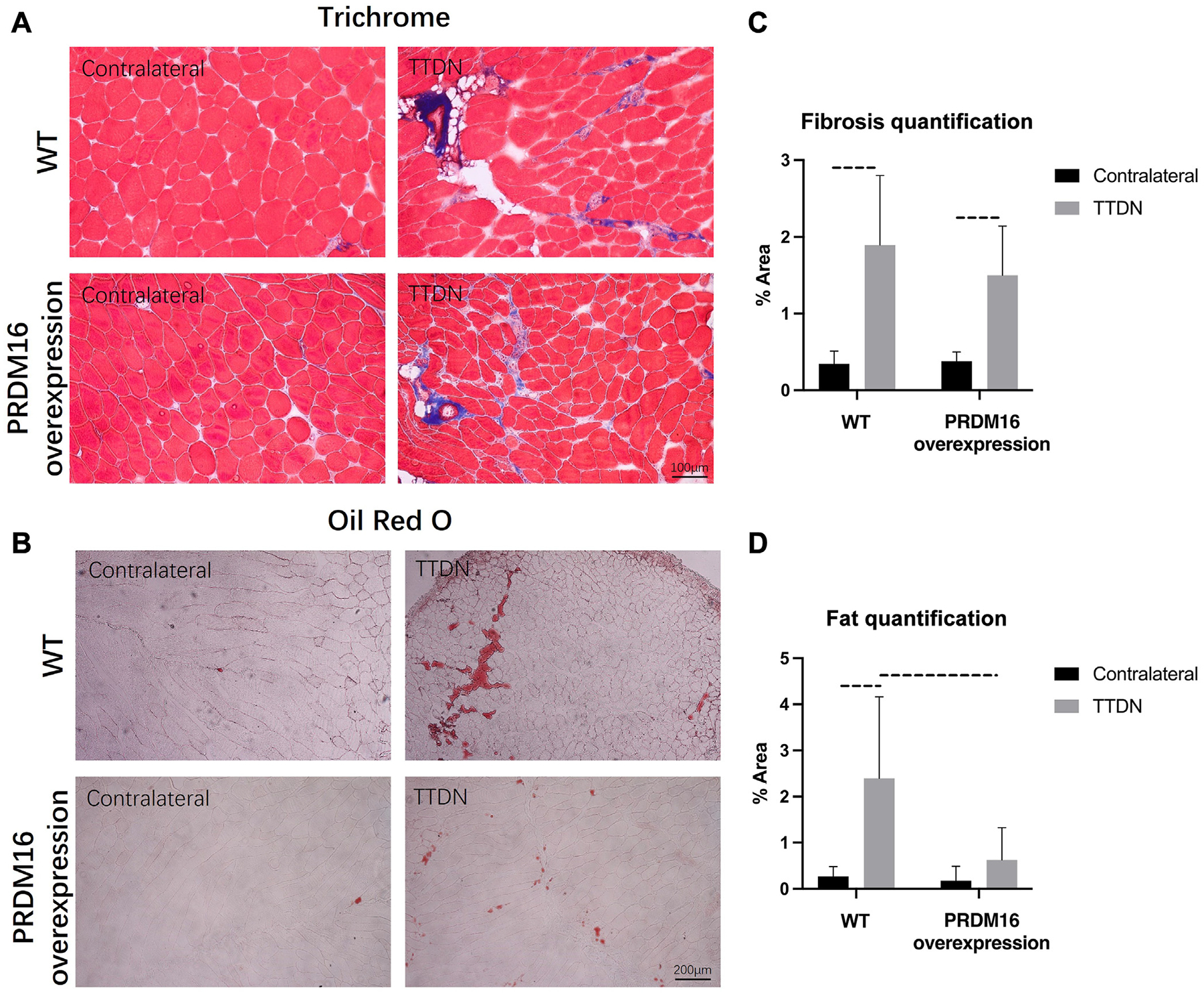
Histologic analysis of supraspinatus muscle in PRDM16-overexpressing and WT mice at 6 weeks after TTDN. (**A**) Representative Masson Trichrome staining images of supraspinatus muscle for muscle fibrosis. (**B**) There was no significant improvement in fibrosis between the PRDM16-overexpressing mice compared with WT in the TTDN supraspinatus muscle. (**C**) Representative Oil Red O staining images of supraspinatus muscle for fatty infiltration. (**D**) PRDM16-overexpressing mice had significantly less fatty infiltration compared with WT mice in the TTDN supraspinatus muscle. WT mice had a significantly greater fatty infiltration in the TTDN supraspinatus muscle than its respective contralateral muscle, whereas PRDM16-overexpressing mice had no significant difference in fatty infiltration between its 2 muscle groups. Quantifications were calculated as fibrotic or fatty tissue area/total muscle cross-section area (%) in supraspinatus and contralateral muscles. *Dash lines* indicate *P* < .01. *WT*, wild type; *TTDN*, right supraspinatus tendon transection and suprascapular nerve transection.

Trichrome staining revealed that mice with PRDM16 overexpression demonstrated no improvement in fibrosis when compared to WT mice after rotator cuff injury (Fig. 2, A). The degree of fibrosis was measured by the percentage of stained collagen fibers per field of view. In WT mice, there was a significant increase in fibrosis in TTDN supraspinatus muscles vs. contralateral muscles (0.347% ± 0.164% vs. 1.895% ± 0.905%, *P* = .002). In PRDM16-overexpressing mice, there was also a significant increase in fibrosis (0.381% ± 0.016% vs. 1.496% ± 0.466%, *P* = .003); however, the percentage of fibrosis between WT and PRDM16-overexpressing mice was not significant (1.548% ± 0.868% vs. 1.115% ± 0.532%, *P* = .320).

### PRDM16 overexpression reduces fatty infiltration of muscle

Oil Red O staining showed that PRDM16 overexpression significantly reduced fatty infiltration after injury (Fig. 2, B). Fatty infiltration was measured by the percentage of area staining for perilipin per field of view. In WT mice, there was a significant increase in the percentage of fatty infiltration in TTDN supraspinatus muscles vs. contralateral muscles (2.389% ± 1.884% vs. 0.233% ± 0.202%, *P* = .015). In PRDM16-overexpressing mice, however, the increase in the percentage of fatty infiltration was not significant (0.639% ± 0.747% vs. 0.186% ± 0.333%, *P* = .190). In supraspinatus muscle after TTDN, fatty infiltration was significantly greater in the WT mice than in the PRDM16-overexpressing mice (2.389% ± 1.884% vs 0.639% ± 0.747%, *P* = .037).

### Enhanced MHC-IIx fiber prevalence in the supraspinatus muscle of PRDM16-overexpressing mice

**Figure 3:**
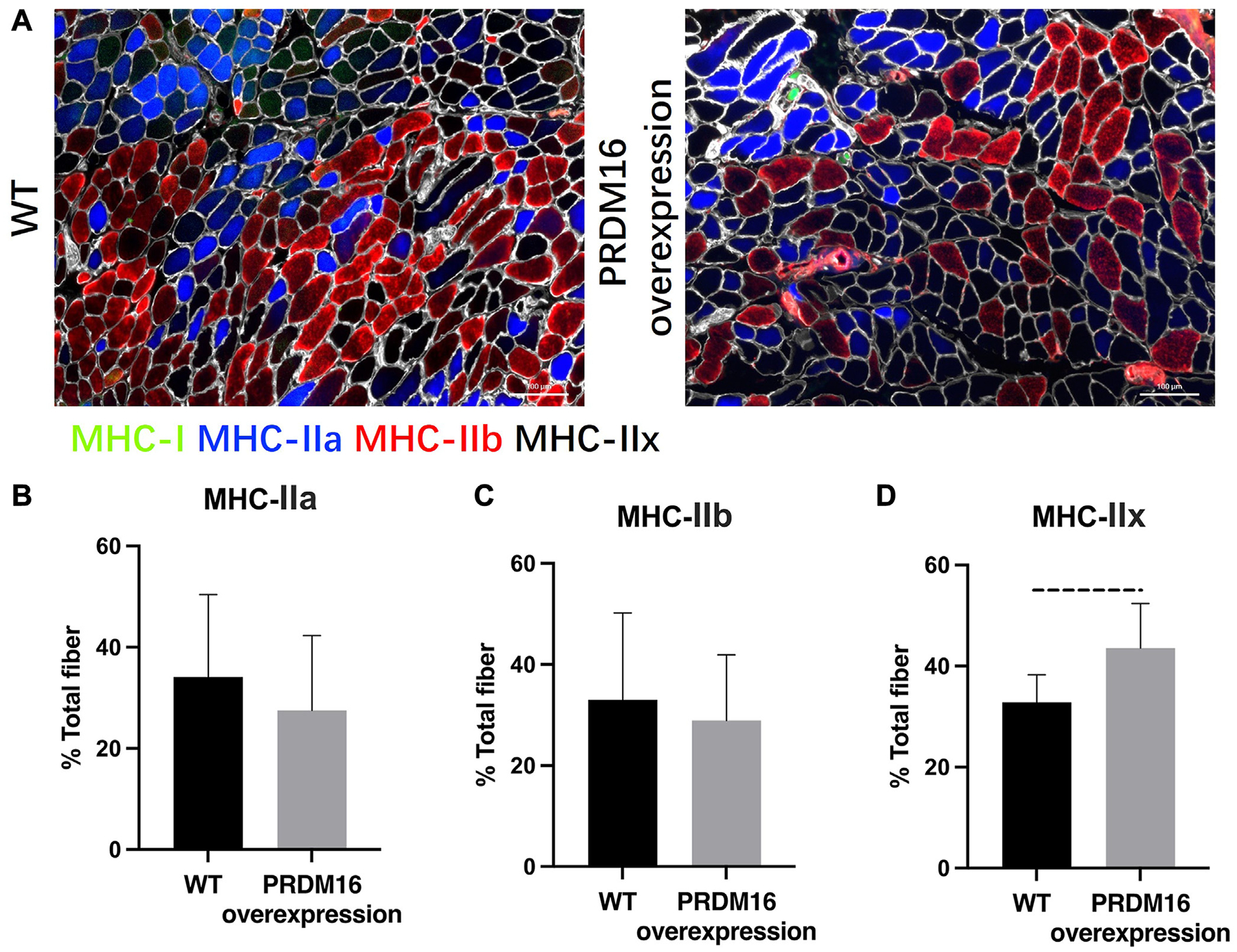
(**A**) Representative images of muscle fiber type staining of supraspinatus muscle in PRDM16-overexpressing and WT mice at 6 weeks after TTDN. (**B-D**) PRDM16-overexpressing mice had significantly increased proportion of MHC-IIx fibers compared with WT mice. There were no significant differences in all other muscle fiber types between the 2 groups. Quantifications were calculated as MHC-IIa, MHC-IIb, or MHC-IIx fibers over total myofibers (%). *Dashed lines* indicate *P* < .01. *WT*, wild type; *TTDN*, right supraspinatus tendon transection and suprascapular nerve transection.

Supraspinatus muscle fibers staining in PRDM16-overexpressing and WT mice groups at 6 weeks post TTDN injury revealed a notable prevalence of MHC-IIx fibers over MHC-IIa and MHC-IIb myofibers (Fig. 3, A). Quantitative analysis using ImageJ demonstrated a significant increase in the proportion of MHC-IIx fibers in the supraspinatus muscle of mice with PRDM16 overexpression compared with the WT mice at 6 weeks post TTDN injury (44.73% vs. 32.84%, *P* = .0053) (Fig. 3, B). Conversely, there were no statistically significant differences observed between PRDM16-overexpressing and WT mice in terms of inducing the percentages of MHC-IIa and MHC-IIb muscle fibers.

## Discussion

In this study, we used our rotator cuff injury mouse model that replicates the fibrosis and fatty infiltration frequently seen in patients with rotator cuff tears to assess the impact of overexpression of PRDM16 on muscle quality and recovery. We have demonstrated that increased expression of PRDM16 promotes decreased fatty infiltration of supraspinatus muscle after rotator cuff injury. Overexpression of PRDM16 also significantly improves shoulder function as detected during gait analysis, indicating the importance of this gene in health outcomes after rotator cuff injury. These data suggest that PRDM16 may be a potential novel therapeutic target for patients with rotator cuff tears.^24^

In the setting of a rotator cuff tear, fatty infiltration and fibrosis of muscle are quite common.^1^ Unfortunately, treatment options are limited to decrease these pathologies once they are present. A study by Gerber et al^9^ in a rabbit rotator cuff tear model demonstrated that the administration of anabolic steroids results in the absence of fatty infiltration on imaging. Another study by Davis et al^7^ found that treatment with simvastatin in rats after full-thickness supraspinatus tear reduced fibrosis by 50%. However, these potential modalities lack target specificity. The resident stem cells in muscle, known as FAPs, have been shown to contribute to fatty infiltration and fibrosis.^14,15,37,45^ However, when FAPs differentiate into BAT-FAP, they have been shown to decrease fibrosis and fatty infiltration, with transplantation of these cells improving outcomes post-injury.^21^ PRDM16 is a critical transcription factor in brown and beige fat differentiation. It plays an important role in white fat “browning.”^33^ Although previous studies have illuminated its significance in metabolic regulation, our research extends its relevance to musculoskeletal health in a rotator cuff tear context.

Previous studies have suggested PRDM16 exerts its positive impact on muscle growth through its dependent interaction with PPARγ as PPARγ is known to be required for adipogenesis.^28,36^ Upregulation of PRDM16 corresponds with other known BAT gene markers including UCP1.^2,32^ These same gene markers are upregulated in response to injury and leads to the differentiation of BAT.^3^ The batokines secreted by BAT including adiponectin, leptin, and lipokines have all been linked to have beneficial effects on muscle growth and prevention of atrophy.^16,29,34^ In regard to white adipose tissue, PRDM16 induces a brown fat tissue phenotype by activating PGC-1α and PGC-1β through direct protein binding.^33^ This decrease in white adipose tissue, coupled with increased muscle regeneration, improves tissue quality and could greatly impact the outcomes of rotator cuff tear, as currently there is a lack of clinically applicable methods to decrease fatty infiltration in these patients.^26^

As a result of our investigation into the overexpression of PRDM16, we found that there was significantly more PRDM16 expression within brown adipose tissue and white adipose tissue within the transgenic mice compared with WT. Furthermore, research suggests that BAT secretes growth factors such as IGF1 and follistatin that promote myogenesis.^38^ The secretion of these factors by BAT may explain the observed improvements we see in forelimb function in PRDM16 overexpression groups compared with the control. However, further research is needed to elucidate the specific biomolecular pathways through which PRDM16 mediates these effects. In addition, the genetic loss of PRDM16 has been shown to mimic the aging effects on muscle quality by promoting fibrosis, whereas additional research in increasing PRDM16 levels in aged mice shows both beige adipose tissue development and amelioration of fibrosis.^40^ In our investigation, we saw no change in fibrosis with PRDM16 overexpression; however, we observed significant improvement in the amount of fatty infiltration. This suggests a pathway exists in which PRDM16 can limit fat accumulation in injured muscle.

It is known that type I fiber–specific atrophy and slow to fast fiber transition is a phenotype of denervation, hence the absence of MHC I fibers in our TTDN injury.^41^ Interestingly, we observed significant induction of the MHC-IIx fiber (fast twitch) in our PRDM16 overexpression model, and this induction has not been detailed in prior literature. Studies discussing the impact of muscle fiber type on muscle recovery and regeneration have primarily used extensor digitorum longus (90% fast-twitch fibers) and soleus muscles (75% slow-twitch fibers).^5,23,30^ Although the extensor digitorum longus is capable of adequate recovery postinjury, soleus muscle regeneration is associated with poor recovery and fibrosis.^4,18^ These studies indicate that large quantities of fast-twitch muscle fibers correlate with improved healing. In our study, overexpression of PRDM16 and muscle type switching correlated with improvement in muscle function, as demonstrated in our gait analysis. Further inquiry is required into the mechanism and relationship between PRDM16 and fiber switching.

Increased PRDM16 through pharmacologic intervention may have clinical utility. As it stands, in rotator cuff tear patients, interventions involving fibroadipogenic progenitor cells may be the key to decrease fatty infiltration and improving muscle quality, thus reducing retear rates.^26^ Methods to target other regulators of the BAT pathway, including upregulation of UCP1 through mirabegron treatment or downregulation of myostatin via soluble activin type IIB receptor, have shown promise in increasing muscle growth and decreasing fat accumulation.^44,48^ Peng et al^27^ have shown that daily injections with l-theanine is one such pathway to activate BAT differentiation through the PRDM16 pathway. Although the side effect of effective dosages of these interventions have not been investigated yet in humans, PRDM16 provides an additional potential therapeutic pathway for muscle regeneration after injury.

This study has several limitations. In the mouse rotator cuff tear model adopted in this study, the torn tendon spontaneously reattaches to the humerus after tendon transection. However, this spontaneous healing does not account for full recovery of forelimb function or improvement in muscle quality without surgical repair as proven in our previous studies with this model.^21,42,43^ To further evaluate the role of PRDM16 in RC muscle recovery after tendon repair, we will consider conducting RC repair on PRDM16-overexpressing mice in our future studies. Our previous studies showed that DigiGait is a reliable method in testing shoulder function in mice after rotator cuff tears and repair; however, motion kinematics of quadrupedal animals may not readily correlate with human patients.^42^ We recently developed a low-cost AI-based string-pulling task to evaluate shoulder function in both humans and mice.^6^ We will consider the use of this string-pulling task for advanced shoulder function testing for mice with rotator cuff tears in the future. Additionally, we only adopted a single time point in this study. More work is needed to see the effects of PRDM16 overexpression in acute rotator cuff tears along with immediate and delayed repair of the rotator cuff. Lastly, we did not investigate FAP differentiation with PRDM16 overexpression. This is to be accomplished in future studies.

## Conclusion

In this study, we found that overexpression of PRDM16 led to improved shoulder function and decreased fatty infiltration in a massive rotator cuff injury mouse model. These findings suggest that promoting a PRDM16 signaling pathway may be used as a novel strategy to enhance the recovery and postsurgical outcomes of patients with rotator cuff tears.
